# Supplementary figures and images for: Antibody profiling and prevalence in US patients during the SARS-CoV2 pandemic
Source: PLoS One. 2020 Nov 19;15(11):e0242655. doi: 10.1371/journal.pone.0242655 (PMC7676701; doi:10.1371/journal.pone.0242655)

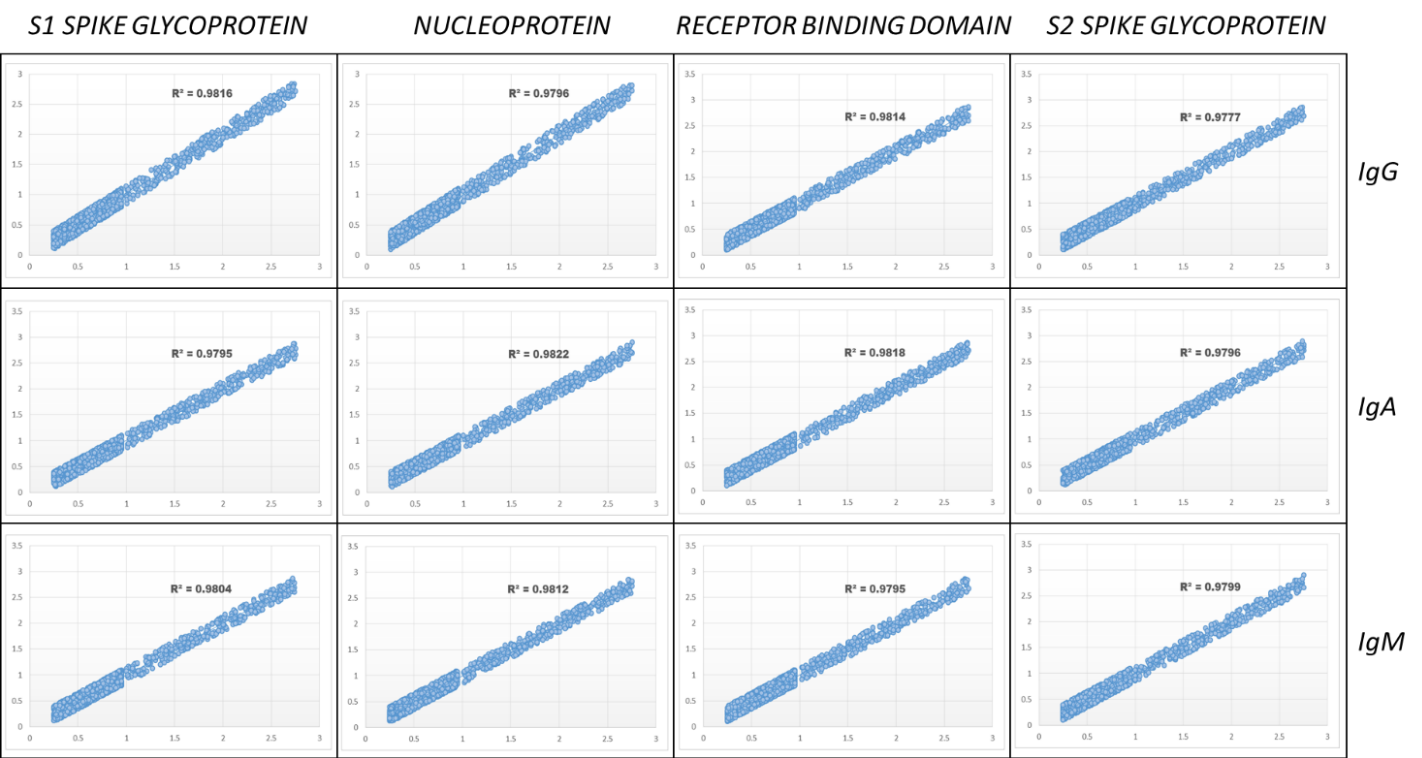

Supplement: S1 Fig — (PNG) [file pone.0242655.s001.png]
